# Precipitation Alleviates Adverse Effects of Nitrogen and Phosphorus Enrichment on Soil Microbial Co-Occurrence Network Complexity and Stability in Karst Shrubland

**DOI:** 10.3390/microorganisms13092012

**Published:** 2025-08-28

**Authors:** Jiangnan Li, Jie Zhao, Xionghui Liao, Xianwen Long, Wenyu Wang, Peilei Hu, Wei Zhang, Kelin Wang

**Affiliations:** 1 College of Environment and Ecology, Hunan Agricultural University, Changsha 410128, China; jiangnan@stu.hunau.edu.cn; 2 Institute of Subtropical Agriculture, Chinese Academy of Sciences, Changsha 410125, China; xhliao@isa.ac.cn (X.L.); longxianwen22@mails.ucas.ac.cn (X.L.); peileihu@isa.ac.cn (P.H.); zhangw@isa.ac.cn (W.Z.); 3 Huanjiang Agriculture Ecosystem Observation and Research Station of Guangxi, Guangxi Key Laboratory of Karst Ecological Processes and Services, Huanjiang Observation and Research Station for Karst Ecosystems, Chinese Academy of Sciences, Huanjiang 547100, Guangxi, China; 4 Guangxi Industrial Technology Research Institute for Karst Rocky Desertification Control, Nanning 530012, China

**Keywords:** karst shrubland, microbial co-occurrence network, nitrogen addition, phosphorous addition, soil microorganism, water addition

## Abstract

The karst region is highly ecologically fragile due to its unique geology and poor water and nutrient retention. Despite long-term restoration, vegetation often remains in the secondary shrubland stage. Soil microorganisms play a vital role in maintaining ecosystem functions, but how microbial communities respond to combined water and nitrogen-phosphorus nutrient changes in karst shrubland remains poorly understood. This knowledge gap hinders effective restoration strategies in karst shrublands. Here, the effects of water, nitrogen, and phosphorous additions and their interactions on soil physico-chemical properties, soil microbial abundance, diversity, community composition, and the co-occurrence network were explored. A full factorial experiment (water × nitrogen × phosphorous, each at two levels) was conducted in a karst shrubland with over 20 years of vegetation restoration, with treatments including control, water (+120 mm yr^−1^), nitrogen (+20 g N m^−2^ yr^−1^), phosphorus (+16 g P m^−2^ yr^−1^), and their four combinations. Our results suggested that water addition significantly increased soil water content and soil microbial abundance but reduced fungal diversity. Nitrogen addition significantly increased soil nitrate nitrogen content and fungal diversity, and fungal diversity showed an increasing trend under phosphorous addition. The addition of nitrogen and phosphorous did not significantly alter the soil microbial community composition, while water addition showed a tendency to change the soil fungal community composition. Network topological properties, robustness, and vulnerability analyses indicated that individual nitrogen or phosphorous additions, as well as their interactions, reduced network complexity and stability. In contrast, water addition alone or in combination with nitrogen and/or phosphorous alleviated these negative effects, and the water and phosphorous interaction exhibited the highest levels of network complexity and stability. Further analysis showed that the soil pH, available phosphorous, ratio of carbon to phosphorous, and ammonium nitrogen were explanatory variables contributing significantly to soil microbial abundance, diversity, community composition, and network complexity. Overall, these findings highlighted the pivotal role of water availability in enhancing soil microbial stability under nutrient enrichment, offering valuable insights into ecological restoration in karst ecosystems.

## 1. Introduction

Soil microorganisms are the most biologically diverse community in terrestrial ecosystems and play key roles in maintaining ecosystem function and stability [1,2,3]. Human-induced global changes, such as warming, alterations in precipitation, the intensification of droughts, and increased atmospheric deposition, have become major drivers shaping soil microbial communities across local and global scales [4,5]. Among these factors, the variation in soil moisture and nutrients is especially critical, as it directly influences microbial metabolism, community structure, and biogeochemical processes [6,7,8]. Understanding how microbial communities respond to shifts in water availability and nutrient inputs is thus essential for predicting ecosystem responses under ongoing climate change.

**Figure 1:**
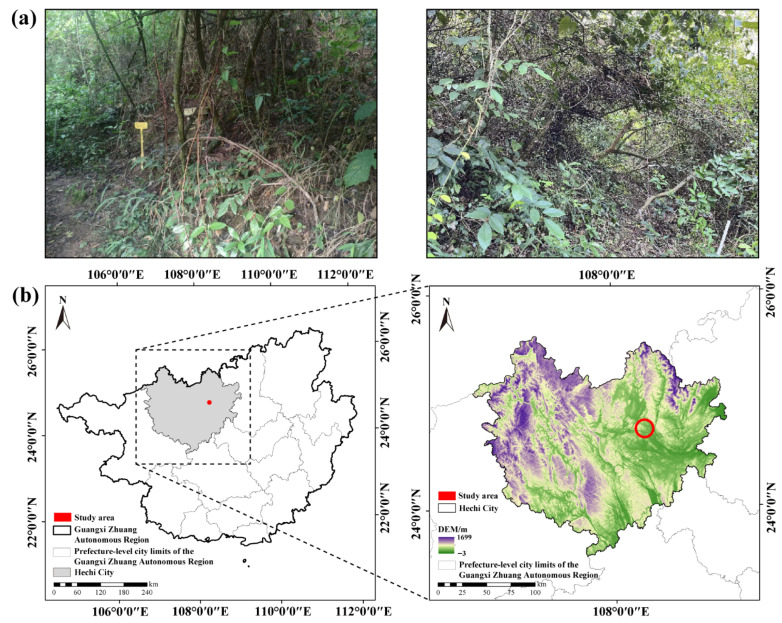
(**a**) Representative landscape of the experimental plots in the karst shrubland, showing typical vegetation and site conditions. (**b**) Location of the study area in the Guangxi Zhuang Autonomous Region, China.

In southwestern China, karst landscapes extend across approximately 540,000 km^2^, accounting for about 5.8% of the country’s total land area [9]. These regions are characterized by extensive bedrock outcrops, well-developed bedrock fractures and underground conduit systems, and the shallow, sparse discontinuity of soils [9,10]. Combined with intense anthropogenic disturbances, these factors have led to the severe co-loss of soil moisture and nutrients, resulting in widespread ecosystem degradation that poses a critical challenge to regional sustainability [9,10,11]. Although decades of ecological restoration have shown progress, large areas remain stalled in shrubland stages (Figure 1a). Previous studies suggest that soil microbial communities in karst shrublands are co-limited by carbon and phosphorous (P) [12,13], yet these findings are mostly based on ecological stoichiometry rather than experimental evidence. Moreover, karst regions exhibit heightened ecological vulnerability under global climate change [14,15]. Water may act as a critical factor modulating nitrogen (N) and P co-limitation and could emerge as a key constraint on microbial communities. However, the effects of water and nitrogen–phosphorus additions and their interactions on soil microbial communities in karst shrublands remain poorly understood.

The direction and magnitude of effects of water and nutrients on soil microbial communities and functions often depend on whether the ecosystem is subjected to water stress or is limited by N and/or P nutrients. For example, in arid ecosystems, increased water availability significantly enhances microbial biomass [16,17]; whereas in humid ecosystems, excessive moisture can saturate pore spaces and suppress the growth of aerobic microorganisms [18]. In N-limited forest ecosystems, long-term N addition has been shown to increase fungal abundance and diversity [19]. Conversely, excessive N inputs can suppress microbial biomass and enzyme activity [20,21], promote the overgrowth of certain microbial groups while inhibiting others, thereby reducing microbial diversity and altering community composition [22,23]. In P-limited tropical forests, P addition significantly increased total microbial and bacterial biomass [24,25], likely due to the high microbial demand for P and enhanced nutrient acquisition capacity. In contrast, in temperate grasslands with weaker P-limited ecosystems, P addition has been found to significantly reduce total microbial and bacterial biomass [26]. In addition, changes in soil moisture and nutrient availability influence plant productivity and carbon allocation, thereby altering the inputs of above- and below-ground litter and indirectly shaping microbial community structure and function [27,28,29,30]. These findings suggest that the impacts of water and nutrient inputs on microbial communities are highly dependent on soil nutrient conditions and may differ substantially under unique edaphic and geological conditions.

Microbial communities are not only characterized by the number and composition of taxa but also by the ecological associations among microorganisms [3]. Evidence is growing that the structural properties of microbial networks, which represent potential interactions among coexisting microorganisms, can strongly influence how communities respond to environmental change and climate extremes [18,31]. Network complexity and stability are important topological properties. Complexity metrics (e.g., number of edges, density, and modularity) describe how many potential associations exist, and stability metrics (e.g., robustness) show how well the network can tolerate species loss [31,32]. Previous studies have shown the effects of water, nitrogen, and phosphorus on the complexity and stability of soil microbial networks. For example, aridity can reduce the stability and complexity of protistan networks by increasing the role of stochastic processes in community assembly and decreasing community-level niche overlap [33]. The N input reduced bacterial network complexity, likely due to changes in bacterial life-history strategies, while having little effect on fungal networks [34]. Karst regions have a unique geological background, and microbial network may response to water and nutrient inputs may differ from those in other ecosystems. 

Here, our goal was to investigate how water and nitrogen–phosphorus nutrient additions affect soil microbial communities and the co-occurrence network in karst shrublands. To achieve this, we conducted a field experiment involving water and nutrient addition treatments and examined their individual and interactive effects on soil physico-chemical properties, microbial abundance, diversity, community composition, and co-occurrence network structures. The specific objectives were to (1) determine whether water and nutrient addition enhance microbial abundance, diversity, and network complexity and stability by alleviating moisture and nutrient limitations, and (2) assess whether the combined application of water and nutrients exerts a stronger influence on microbial community structure and network stability than either factor alone, due to potential synergistic effects on resource availability.

## 2. Materials and Methods

### 2.1. Site Description and Experimental Design

The study area was conducted at the Huanjiang Observation and Research Station for Karst Ecosystems (107°51′–108°43′ E, 24°44′–25°33′ N), Chinese Academy of Sciences, Guangxi Province, China. This region has a subtropical monsoon climate with a distinct wet season (from April to August) and a distinct dry season (from September to March). The mean annual temperature is 19.6 °C, and the mean annual rainfall is 1389.1 mm. The soils in the area are calcareous soils and developed from a dolostone base [35].

The water, nitrogen, and phosphorous addition experiment was established in May 2022. The experimental platform was established in a typical karst shrubland with over 20 years of vegetation restoration. The dominant vegetation includes various shrub species such as *Alangium chinense*, *Smilax china*, *Zanthoxylum echinocarpum*, *Rubus lambertianus*, and *Vitex negundo*. A randomized block design was employed, with each factor set at two levels (addition vs. no addition), resulting in eight treatments (2 × 2 × 2), including control (C), water addition (W), nitrogen addition (N), phosphorus addition (P), water and nitrogen addition (WN), water and phosphorus addition (WP), nitrogen and phosphorus addition (NP), and water, nitrogen, and phosphorus addition (WNP). The experiment consisted of five blocks, each containing eight 5 m × 5 m plots randomly assigned to the eight treatments. To minimize interference between plots, a 2 m buffer zone was established between adjacent plots. For the water addition treatment, a total of 120 mm m^−2^ yr^−1^ was applied in two equal increments (60 mm m^−2^ each) during periods of prolonged drought (≥14 consecutive rainless days) between April to June and July to September. Nitrogen and phosphorus were added at annual rates of 20 g N m^−2^ yr^−1^ and 16 g P m^−2^ yr^−1^, respectively, split into two equal applications in April and July. Ammonium nitrate (NH_4_NO_3_) and sodium dihydrogen phosphate dihydrate (NaH_2_PO_4_·2H_2_O) were used as N and P sources, respectively. For each plot, the fertilizers were dissolved in 50 L of water and uniformly sprayed onto the soil surface using a 20 L backpack sprayer (3WBD-20L, LanYi, Suqian China). Spraying was conducted carefully to avoid surface runoff, and no visible runoff was observed during the application.

### 2.2. Soil Sampling

Soil samples were collected at a depth of 0–10 cm in July 2023. An auger with a 2.5 cm diameter was used to randomly harvest five soil cores from each plot, producing a total of 40 composite samples. In the laboratory, visible plant roots and stones in each sample were taken out, and new gloves had to be used after handling a different sample to avoid cross-contamination. Each sample was divided into three subsamples. A subsample (~20 g) was stored at −80 °C before soil microbial DNA extraction. Another subsample (~300 g) was kept at 4 °C was used for analysis of soil water content (SWC), ammonium nitrogen (NH_4_^+^), and nitrate nitrogen (NO_3_^−^). The remanent subsample was air-dried at room temperature for analysis of soil pH, soil organic carbon (SOC), total nitrogen (TN), total phosphorus (TP), available phosphorus (AP), exchangeable calcium (Ca^2+^), and exchangeable magnesium (Mg^2+^).

### 2.3. Soil Physico-Chemical Properties Analysis

Soil water content (SWC) was measured by oven-drying for 12 h at 105 °C. Soil pH was determined in 1:2.5 (*w*/*v*) soil solutions using a pH meter (FE20K, Mettler-Toledo, Greifensee, Switzerland). Soil ammonium (NH_4_^+^) and nitrate nitrogen (NO_3_^−^) were extracted with 2 mol L^−1^ KCl and determined with a continuous flow analyzer (Auto Analyzer 3-AA3, Seal Analytic, Mequon, GA, USA) [36]. SOC was determined by wet oxidation using the dichromate redox colorimetric method [37]. The Kjeldahl method measured soil total nitrogen (TN) [38]. Soil total phosphorus (TP) and available phosphorus (AP) were determined by the molybdenum antimony colorimeter method after digestion in HClO_4_-H_2_SO_4_ (1:10, *v*/*v*) and extraction in NaHCO_3_ (0.5 mol L^−1^), respectively [39]. Soil total potassium (TK) and available potassium (AK) were measured by the atomic absorption method after being digested in NaOH and extracted in NH_4_OAc (1.0 mol L^−1^), respectively [40]. Soil exchangeable calcium (Ca^2+^) and exchangeable magnesium (Mg^2+^) were displaced via compulsive exchange in ammonium acetate (1 mol L^−1^) and were then analyzed using an inductively coupled plasma emission spectrometer (5110 ICP-OES; Agilent, Santa Clara, CA, USA) [41].

### 2.4. Gene Extraction, Replication, and Sequencing

Soil DNA was extracted using a Fast DNA^®^ SPIN Kit (MoBio Laboratories, Carlsbad, CA, USA) according to the manufacturer’s instructions. Real-time quantitative PCR (qPCR) was performed to quantify the soil bacterial and fungal abundance (gene copy number) using the quantitative PCR system for ABI 7300 (Applied Biosystems, Waltham, MA, USA). The primer sets Eub338 (5′–ACTCCTACGGGAGGCAGCAG–3′) and Eub806 (5′–GGACTACHVGGGTWTCTAAT–3′), and ITS1F (5′–CTTGGTCATTTAGAGGAAGTAA–3′) and ITS2R (5′–GCTGCGTTCTTCATCGATGC–3′) were used to amplify the V3–V4 region of 16S rRNA and the ITS1 region of 18S rRNA genes, respectively [42,43]. The amplification system was as follows: 4 μL of Taq Plus Master Mix (2×), 0.8 µL of each forward and reverse primer (5 µM), 2 µL of the DNA template, and 7.2 µL of ddH_2_O. The qPCR reaction conditions were maintained at initial denaturation at 95 °C for 5 min, followed by 35 cycles of 95 °C for 30 s (denaturation), 58 °C for 30 s (annealing), and final extension at 72 °C for 1 min. The 16S rRNA and 18S rRNA gene amplifications exhibited efficiencies of 92.12% and 104% and R^2^ values of 0.998 and 0.999, respectively.

Bacterial and fungal communities were determined by 16S and ITS gene amplicon sequencing, respectively. The bacterial 16S rRNA (515F, 5′–GTGCCAGCMGCCGCGG–3′/907R, and 5′–CCGTCAATTCMTTTRAGTTT–3′) and fungal ITS rRNA (ITS1F, 5′–CTTGGTCATTTAGAGGAAGTAA–3′/ITS2R, and 5′–GCTGCGTTCTTCATCGATGC–3′) sequences were amplified using an ABI GeneAmp^®^ 9700 PCR thermocycler (ABI, Oakland, CA, USA) [43,44]. The PCR mixtures (20 μL) consisted of 10 μL of Pro Tap (2×), 0.8 μL of forward primer (5 μM), 0.8 μL of reverse primer (5 μM), 10 ng of the template DNA, and sterile ddH_2_O to a final volume of 20 µL. The PCR amplification was performed as follows: initial denaturation for 3 min at 95 °C; followed by denaturing at 95 °C for 30 s and annealing at 55 °C for 30 s; an extension at 72 °C for 45 s (27 cycles for 515F/907R and 35 cycles for ITS1F/ITS2R); and a single extension at 72 °C for 10 min. PCR products were extracted from 2% agarose gel and purified using the AxyPrep DNA Gel Extraction Kit (Axygen Biosciences, Union City, CA, USA), according to the manufacturer’s instructions. Purified amplicons were pooled in equimolar concentrations and paired-end sequenced on an Illumina MiSeq PE300 platform (Illumina, San Diego, CA, USA), according to the standard protocols by Majorbio Bio-Pharm Technology Co., Ltd. (Shanghai, China).

The raw sequencing reads were first processed using fastp (version 0.20.0; https://github.com/OpenGene/fastp (accessed on 18 January 2024)) for quality control, including adapter trimming, quality filtering, and the removal of reads shorter than 50 bp. A total of 2,629,046 and 3,854,167 raw reads were generated for the 16S rRNA gene and ITS rRNA gene, respectively, of which 2,523,865 (16S) and 3,430,727 (ITS) high-quality reads remained after quality filtering. Subsequently, paired-end reads were merged using FLASH (version 1.2.7; http://www.cbcb.umd.edu/software/flash (accessed on 18 January 2024)) [45,46]. The quality-filtered sequences were then clustered into operational taxonomic units (OTUs) at a 97% similarity threshold using UPARSE (version 7.1; http://drive5.com/uparse/ (accessed on 18 January 2024)) [47], resulting in 3157 OTUs for the 16S rRNA gene and 739 OTUs for the ITS rRNA gene after removing chimeras. Taxonomic classification was performed using the RDP classifier (version 2.2; http://rdp.cme.msu.edu/ (accessed on 18 January 2024)) with a confidence threshold of 0.7, which is the default and commonly recommended value to balance taxonomic resolution and classification accuracy [48]. Reference databases were selected according to microbial groups: SILVA 138 for bacteria and UNITE 8.0 for fungi. The sequences of bacteria and fungi have been submitted to the NCBI Sequence Read Archive (SRA) with accession number SRP607826 and SRP607847.

### 2.5. Co-Occurrence Network Analysis

Co-occurrence network analysis was performed to explore the potential paired species associations by applying the SparCC method implemented in ggClusterNet package (version 0.1.0) [49]. To reduce the influence of rare taxa on the correlation analysis and to simplify network complexity, OTUs with an abundance greater than 400 were selected. Robust associations were identified under a correlation threshold of 0.6 and a significance level of 0.01, resulting in the construction of a non-random, multitrophic-level network [50]. Pseudo *p*-values were calculated based on 100 bootstrap iterations, and robust associations were further screened according to the aforementioned thresholds. Using the subgraph function in the igraph package, OTUs present in individual samples were retained to extract sub-networks specific to each sample. Topological properties were then calculated for each sub-network, including the number of edges, positive correlation ratio, negative correlation ratio, number of vertices, graph density, average degree, and relative modularity. Higher values of the edge number, average degree, and graph density indicate a denser connectivity and greater network complexity [50,51]. The patchwork and pulsar packages were employed to calculate the robustness and vulnerability of the co-occurrence networks in order to evaluate network stability [32,50]. A greater robustness indicates stronger network stability, whereas a greater vulnerability reflects lower stability and higher susceptibility to disturbances [52].

### 2.6. Statistical Analysis

The Shannon index was used to assess microbial diversity and was calculated using R package vegan (version 2.6-8) [53]. All data were tested for normal distribution and variance homogeneity before analysis, and the ln (x + 1) transformation was performed when required to meet the criteria. A one-way ANOVA with the Least Significant Difference (LSD) test was utilized to identify significant differences (*p* < 0.05) in soil physico-chemical properties, microbial abundance and diversity, and topological properties of soil microbial co-occurrence networks. A multiway-ANOVA (MANOVA) was employed to examine the effects of water, nitrogen, phosphorus, and their interactions on the above indicators (*p* < 0.05). To evaluate the effects of different treatments on soil bacterial and fungal community composition, non-metric multidimensional scaling (NMDS) based on Bray–Curtis dissimilarity matrices was performed using the metaMDS function in the vegan package [53]. The significance of treatment effects was further assessed by analysis of similarities (ANOSIM). A mantel test was conducted to evaluate relationships between the microbial community composition and soil physico-chemical properties using the R package linkET (version 0.0.7.4) [54,55]. We also used RDA with forward selection to select the explanatory soil properties that contributed significantly (*p* < 0.05) to the variation in soil microbial abundance, diversity, community composition, and co-occurrence network complexity [56]. In the RDA, the microbial community composition was based on the NMDS analysis, with the data for each sample derived from NMDS1. The RDA was carried out using Canoco 5.0 (Centre for Biometry, Wageningen, The Netherlands). The statistical significance of RDA results was determined using the Monte Carlo permutation method, based on 999 runs with randomized data.

## 3. Results

### 3.1. Soil Physico-Chemical Properties

The three-way ANOVA results showed that the W addition significantly increased SWC by 7.58% (*p* < 0.05), the N addition significantly increased soil NO_3_^−^ content by 57.66% (*p* < 0.01), and the P addition significantly increased the soil C:N ratio by 16.09% (*p* < 0.05, Table 1 and Table S1). Moreover, a significant interactive effect between the N and P addition was observed, which further increased the soil C:N ratio (*p* = 0.009, Table 1 and Table S1). Subsequent one-way ANOVA results revealed more detailed effects of specific treatments on the soil C:N ratio and NO_3_^−^ content (*p* < 0.05, Table 1). Specifically, the soil C:N ratio in the NP treatment was significantly higher than in the C, N, P, and WN treatments (*p* < 0.05, Table 1). Additionally, the soil C:N ratio in the W and WNP treatments was significantly higher than in the N and P treatments (*p* < 0.05, Table 1). For soil NO_3_^−^ content, the WN treatment resulted in significantly higher levels compared to the C, W, P, and WP treatments (*p* < 0.05, Table 1).

### 3.2. Soil Microbial Abundance and Diversity

**Figure 2:**
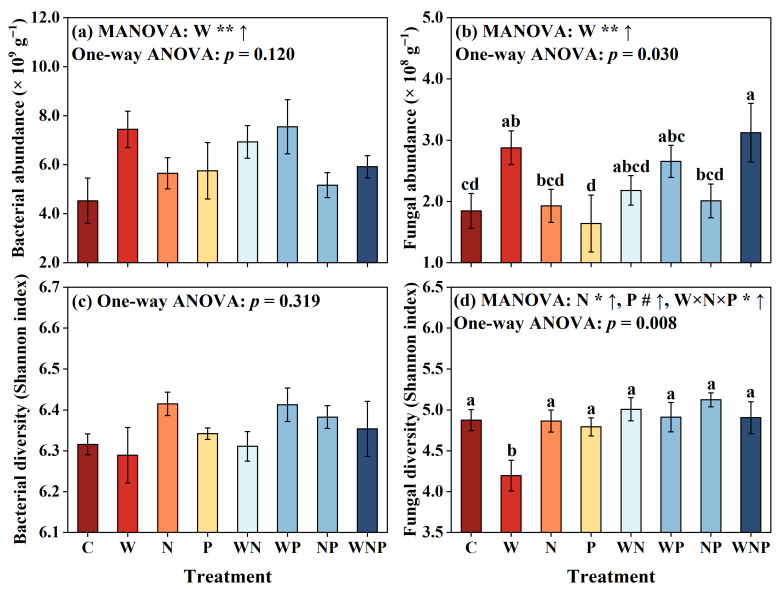
Effects of water (W), nitrogen (N), and phosphorus (P) additions and their interactions on the abundance and diversity of soil bacteria (**a**,**c**) and fungi (**b**,**d**). MANOVA results are shown above each panel and reflect the effects of individual and interactive factors. The “↑” and “↓” symbols indicate a significant increase and decrease compared to the control based on MANOVA, respectively. Significance levels at # *p* < 0.1, * *p* < 0.05, and ** *p* < 0.01. Different lowercase letters above the bars indicate significant differences among treatments (*p* < 0.05), as determined by one-way ANOVA followed by LSD tests. C, control; W, water addition; N, nitrogen addition; P, phosphorus addition; WN, water and nitrogen addition; WP, water and phosphorus addition; NP, nitrogen and phosphorus addition; and WNP, water, nitrogen, and phosphorus addition.

The results of three-way ANOVA revealed that the W addition significantly increased the abundance of soil bacteria and fungi (*p* < 0.01, Figure 2a,b), while the N addition significantly increased the diversity of fungi (*p* < 0.05, Figure 2d). Additionally, the interaction of W, N, and P significantly enhanced fungal diversity (*p* < 0.05, Figure 2d). One-way ANOVA results further demonstrated significant effects of different treatments on both soil fungal abundance and diversity. Regarding soil fungal abundance, the WNP treatment was significantly higher than the C, N, P, and NP treatments, the W treatment was significantly higher than the C and P treatments, and the WP treatment was significantly higher than the P treatment (*p* < 0.05, Figure 2b). However, in terms of fungal diversity, the W treatment was significantly lower than the other treatments (*p* < 0.01, Figure 2d).

### 3.3. Soil Microbial Community Composition

**Figure 3:**
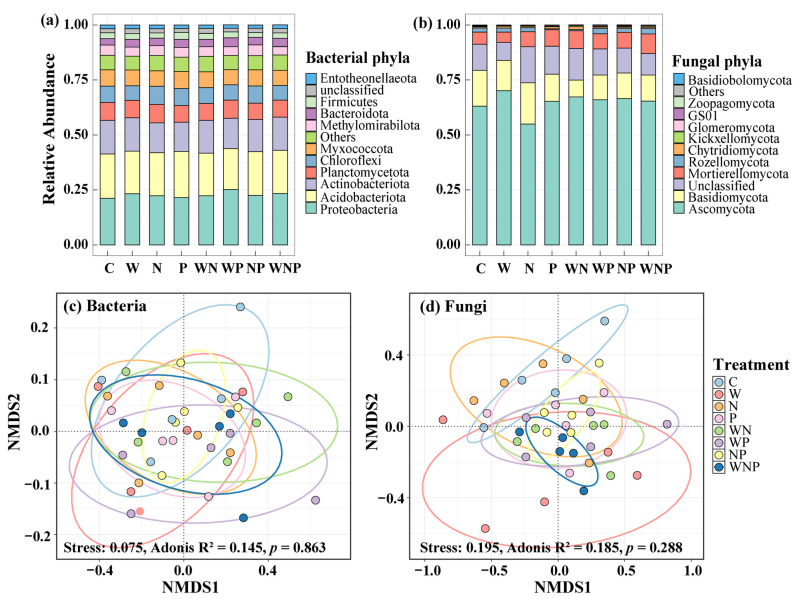
Effects of different treatments on soil bacterial and fungal community composition at phylum level (**a**,**b**), and non-metric multidimensional scaling (NMDS) ordination patterns (**c**,**d**). C, control; W, water addition; N, nitrogen addition; P, phosphorus addition; WN, water and nitrogen addition; WP, water and phosphorus addition; NP, nitrogen and phosphorus addition; and WNP, water, nitrogen, and phosphorus addition.

At the phylum level, the dominant bacterial phyla included *Proteobacteria* (21.2–25.2%), *Acidobacteriota* (18.6–21.0%), *Actinobacteriota* (13.3–15.4%), *Planctomycetota* (7.4–8.4%), *Chloroflexi* (6.6–8.4%), *Myxococcota* (6.4–7.8%), and *Methylomirabilota* (6.9–7.6%, Figure 3a). The dominant fungal phyla consisted of *Ascomycota* (54.9–70.1%), *Basidiomycota* (7.6–18.8%), and *Mortierellomycota* (4.6–8.9%, Figure 3b). An NMDS analysis revealed that different treatments did not significantly alter the community composition of soil bacteria and fungi (Figure 3c,d). However, compared to the control treatment, the W treatment exhibited a noticeable trend of segregation in fungal community composition (Figure 3d). LEfSe analysis showed that the relative abundance of *Nitrospirota* increased in the WNP treatment, while *Latescibacterota* was more abundant in the N treatment (Figure S1a). Additionally, the relative abundance of *Glomeromycota* increased in the NP treatment (Figure S1b).

### 3.4. Soil Microbial Co-Occurrence Network

**Figure 4:**
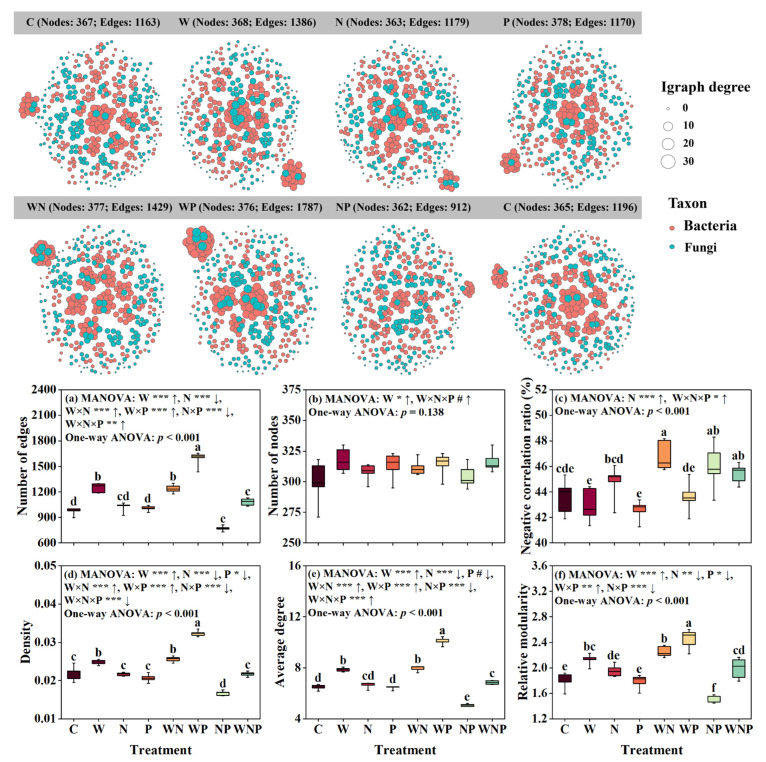
Effects of water (W), nitrogen (N), and phosphorus (P) additions and their interactions on soil microbial co-occurrence network patterns and network topological properties. Network topological properties include the number of edges (**a**), number of nodes (**b**), negative correlation ratio (**c**), density (**d**), average degree (**e**), and relative modularity (**f**). MANOVA results are shown above each panel and reflect the effects of individual and interactive factors. The “↑” and “↓” symbols indicate significant increase and decrease compared to the control based on MANOVA, respectively. Significance levels at # *p* < 0.1, * *p* < 0.05, ** *p* < 0.01, and *** *p* < 0.001. Different lowercase letters above the bars indicate significant differences among treatments (*p* < 0.05), as determined by one-way ANOVA followed by LSD tests. C, control; W, water addition; N, nitrogen addition; P, phosphorus addition; WN, water and nitrogen addition; WP, water and phosphorus addition; NP, nitrogen and phosphorus addition; and WNP, water, nitrogen, and phosphorus addition.

On average, the WP treatment exhibited the highest number of edges, density, average degree, and relative modularity, while the NP treatment had the lowest values (Figure 4a,d–f). The three-way ANOVA results revealed that the W addition and its interactions with N or P significantly increased the number of edges, density, and average degree of the co-occurrence network (*p* < 0.001, Figure 4a,d,e). In contrast, the N and P addition and their interaction significantly decreased these topological properties (*p* < 0.1, Figure 4a,d,e). Furthermore, the interaction between W, N, and P significantly increased the number of edges and nodes, the negative correlation ratio, and average degree of the co-occurrence network (*p* < 0.1, Figure 4a–c,e) but reduced network density (*p* < 0.001, Figure 4d). In addition, the N addition and its interactions with W and P significantly increased the negative correlation ratio (*p* < 0.05, Figure 4c). The W addition and its interactions with P significantly increased the relative modularity (*p* < 0.01), while the N and P addition and their interaction significantly decreased the relative modularity (*p* < 0.05, Figure 4f).

The results of a one-way ANOVA further revealed the specific effects of individual treatments on network topological properties. Compared with the control treatment, the W, WN, and WP treatments significantly increased the number of edges, density, average degree, and relative modularity, while the NP treatment led to a significant reduction in these properties (*p* < 0.001, Figure 4a,d–f). In addition, the WN, NP, and WNP treatments significantly increased the negative correlation ratio compared to the control treatment (*p* < 0.001, Figure 4c).

**Figure 5:**
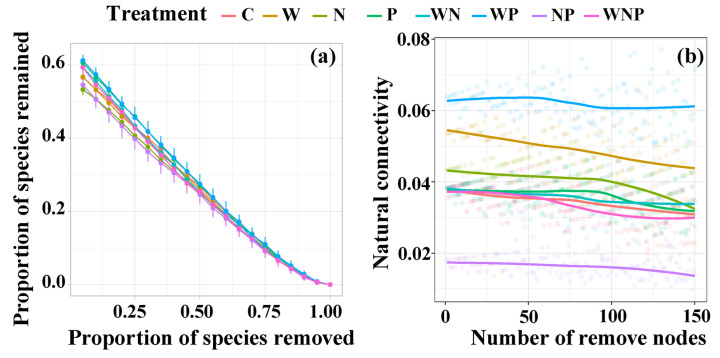
(**a**) Robustness analysis is shown as the relationship between the proportion of species remaining and the proportion of species removed. (**b**) Vulnerability analysis is shown as the relationship between microbial natural connectivity and the number of removed nodes. C, control; W, water addition; N, nitrogen addition; P, phosphorus addition; WN, water and nitrogen addition; WP, water and phosphorus addition; NP, nitrogen and phosphorus addition; and WNP, water, nitrogen, and phosphorus addition.

Compared to the control treatment, the network robustness and vulnerability of the WP, W, N, and P treatments exhibited varying degrees of increases (Figure 5). The greatest increase was observed in the WP treatment, followed by the W treatment, and then the N and P treatments (Figure 5). In contrast, the network robustness and vulnerability of the NP and WNP treatments were lower than those of the control treatment, with the most significant decrease observed in the NP treatment (Figure 5).

### 3.5. Factors Affecting Soil Microbial Communities

**Figure 6:**
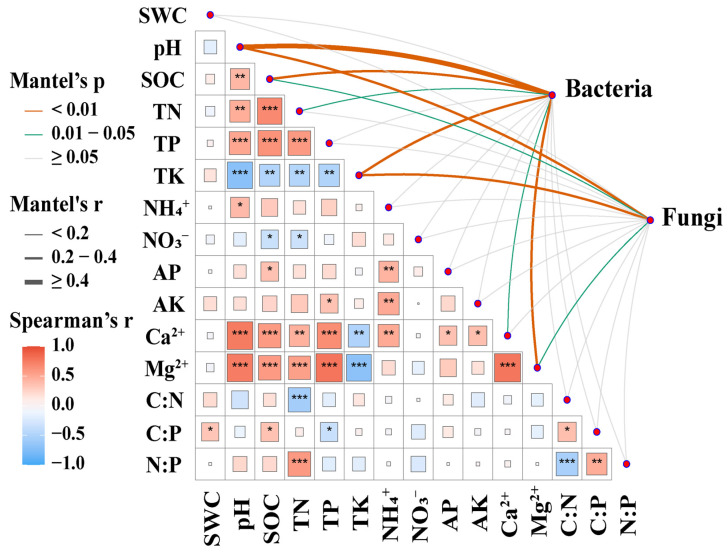
Correlations between the community composition of soil microorganisms and soil physico-chemcial properties. Pairwise comparisons of environmental factors are shown, with a color gradient denoting Spearman’s correlation coefficient. Microbial community composition was related to each soil factor by partial (geographic distance-corrected) Mantel tests. Edge width corresponds to the Mantel’s r statistic for the corresponding distance correlations, and edge color denotes the statistical significance based on 999 permutations. Significant correlations are indicated by symbols (* *p* < 0.05; ** *p* < 0.01; *** *p* < 0.001). SWC, soil water content; pH, soil pH; SOC, soil organic carbon; TN, soil total nitrogen; TP, soil total phosphorus; TK, soil total potassium; NH_4_^+^, soil ammonium nitrogen; NO_3_^−^, soil nitrate nitrogen; AP, available phosphorus; AK, available potassium; Ca^2+^, exchangeable calcium; Mg^2+^, exchangeable magnesium; C:N, soil carbon to nitrogen ratio; C:P, soil carbon to phosphorus ratio; and N:P, soil nitrogen to phosphorus ratio.

**Figure 7:**
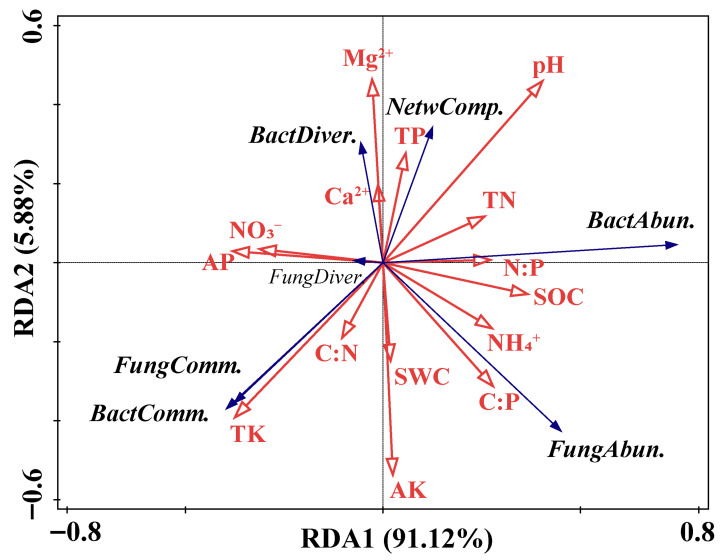
Redundancy analysis with forward selection of microbial abundance, diversity, community composition, and co-occurrence network complexity constrained by the significant (*p* < 0.05) explanatory variables. SWC, soil water content; pH, soil pH; SOC, soil organic carbon; TN, soil total nitrogen; TP, soil total phosphorus; TK, soil total potassium; NH_4_^+^, soil ammonium nitrogen; NO_3_^−^, soil nitrate nitrogen; AP, available phosphorus; AK, available potassium; Ca^2+^, exchangeable calcium; Mg^2+^, exchangeable magnesium; C:N, soil carbon to nitrogen ratio; C:P, soil carbon to phosphorus ratio; N:P, soil nitrogen to phosphorus ratio; *BactAbun.*, bacterial abundance; *FungAbun.*, fungal abundance; *BactDiver.*, bacterial diversity; *FungDiver.*, fungal diversity; *BactComm.*, bacterial community composition; *FungComm.*, fungal community composition; and *NetwComp.*, co-occurrence network complexity.

The results of the Mantel analysis revealed that soil pH, SOC, TN, TK, Ca^2+^, and Mg^2+^ contents were key driving factors influencing the composition of the soil bacterial community (Figure 6). Soil pH, SOC, TK, and Mg^2+^ contents were identified as crucial factors regulating the composition of the soil fungal community (Figure 6). An RDA with forward selection indicated that soil pH, AP, C:P, and NH_4_^+^ were explanatory variables contributing significantly to soil microbial abundance, diversity, community composition, and network complexity (Figure 7). Specifically, bacterial and fungal abundance was positively correlated with soil pH, C:P, and NH_4_^+^ (Figure 7). Soil microbial diversity was positively correlated with soil AP (Figure 7). Furthermore, the complexity of the microbial co-occurrence network was positively correlated with soil pH and negatively correlated with soil AP, C:P, and NH_4_^+^ (Figure 7).

## 4. Discussion

### 4.1. Soil Microbial Abundance Was Sensitive to Water Addition

To determine whether water and nutrient additions enhance microbial abundance and diversity by alleviating resource limitations (Objective I), we measured soil physico-chemical properties and microbial abundance and diversity. The results showed that water addition significantly increased soil water content and promoted the abundance of soil bacteria and fungi (Table 1 and Figure 1). This was primarily due to the direct alleviation of microbial water stress and the improvement of their habitat conditions. The increased soil moisture enhanced soil microbial mobility and substrate diffusion capabilities, thereby facilitating their growth and reproduction [57,58]. Meanwhile, the increased soil moisture accelerated the decomposition of organic matter, releasing more available carbon sources and nutrients, further supporting the expansion of the microbial community [59]. In addition, the increased soil moisture may further stimulate microbial growth by increasing the abundance of predators (e.g., soil nematodes) and the trophic cascade effects between predators and microbes [60,61,62]. This results also indicated that during the karst shrubland stage, water availability is the key limiting factor for soil microbial activities. In addition, the W addition led to a decrease in the relative abundance of *Glomeromycota* (Figure 3). As obligate symbiotic arbuscular mycorrhizal fungi (AMF), *Glomeromycota* heavily depend on carbon sources provided by host plant roots [63]. The increase in soil moisture may inhibit their growth in two ways. On the one hand, increased soil moisture can limit oxygen diffusion and create hypoxic conditions, which may inhibit AMF hyphal growth and spore germination, as they are strictly aerobic [64]. On the other hand, when soil moisture relieves plant drought stress, the host may invest less carbon in mycorrhizal symbiosis and prioritize its own growth [65,66]. Furthermore, the W addition promoted the rapid proliferation of saprophytic fungi and bacteria (e.g., *Ascomycota*, Figure 3b). These microorganisms not only compete with AMF for limited nutrients and ecological niches, but their relative abundance increases statistically, thereby diluting the proportion of AMF.

Our results demonstrated that the N addition significantly increased soil nitrate content and fungal diversity (Table 1 and Figure 2d), while the P addition also showed a trend of increasing fungal diversity (Figure 2d). These findings indicate that nutrient input can alleviate resource limitations that constrain fungal communities in karst shrublands. The increase in fungal diversity under N addition may be attributed to improved N availability, which supports the growth of various fungal taxa, especially saprotrophs and symbiotic species [67]. Under P addition, although the effect on fungal diversity was weaker, the observed trend still suggests that some fungal taxa, such as P-solubilizing fungi, may benefit from the additional P [68,69]. Furthermore, the N addition significantly increased the relative abundance of *Nitrospirota* (Figure 3a and Figure S1a), which are responsible for converting ammonium to nitrate through nitrification [70,71]. This result further supports the result that N addition increased the soil nitrate content (Table 1). In contrast, bacterial abundance and diversity remained largely unresponsive to N and P additions, highlighting a potential difference in how fungi and bacteria respond to nutrient inputs. The reason may be that bacteria have a higher metabolic flexibility and tolerance to changes [72], whereas fungi are more tightly linked to nutrient dynamics and plant interactions [73], making them more sensitive to nutrient enrichment.

### 4.2. Opposing Roles of Water and Nutrients in Microbial Network Complexity and Stability

Our results revealed that N and P additions, particularly their combined addition, reduced the complexity and stability of microbial co-occurrence networks (Figure 4 and Figure 5). This result was contrary to our expectation. There are several possible explanations. First, excess nutrients may lead to ecological imbalance by favoring copiotrophs, which outcompete oligotrophs and reduce the overall network diversity and connectivity [72,74]. Our finding that the complexity of the microbial co-occurrence network was negatively correlated with the available phosphorus, carbon-to-phosphorus ratio, and ammonium nitrogen also supports this theory (Figure 7). Second, in nutrient-poor karst soils, microbes are adapted to oligotrophic conditions and depend on mutualistic relationships [75,76,77]. Third, the combined addition of N and P may lower functional complementarity and redundancy among microbes, weakening the cooperative links that support network stability [78,79].

In contrast, the W addition increased the complexity and stability of microbial networks and mitigated the negative effects of nutrient inputs (Figure 4 and Figure 5). This finding highlights the key role of water as a limiting factor in karst systems. First, in karst systems, high infiltration rates can increase nutrient leaching [80,81], which may dampen the effects of added nitrogen and phosphorus. The rapid water infiltration in karst soils carries away excess nutrients, reducing their availability to microbes and mitigating the nutrient-induced stress on microbial networks. In addition, high nitrogen and phosphorus inputs can lead to a decrease in soil pH [82]. In our study, however, the complexity of the soil microbial network was positively correlated with the soil pH (Figure 7). This also supports the idea that the water addition helps maintain the soil pH by leaching excess nutrients, thereby sustaining microbial network complexity. Previous studies have shown that precipitation during the rainy season can alleviate the adverse impacts of high nitrogen inputs on soil nematode communities [83]. Second, increased water availability reduces microbial stress in dry soils, stimulating microbial activity and interactions [84,85]. Third, water improves nutrient transport and accessibility, enabling plants to absorb and transform nutrients more efficiently [86], while reducing competition among different microbial groups and promoting more effective interactions. Therefore, under conditions of high nutrient input, water addition helps to sustain a more connected and stable microbial network in karst shrublands.

### 4.3. Research Implications

This study demonstrates that water availability plays a pivotal role in mitigating the negative impacts of nitrogen and phosphorus enrichment on soil microbial co-occurrence network complexity and stability in karst shrublands. These findings have direct implications for the conservation and restoration of degraded karst ecosystems.

Karst landscapes are highly vulnerable to water loss due to their shallow soils and high permeability [9], which in turn exacerbates nutrient limitations and destabilizes microbial communities. Chemical nitrogen and phosphorus fertilizers are often used in restoration practices to overcome nutrient deficiencies and promote rapid plant establishment [87,88]. However, excessive chemical fertilization can disrupt microbial symbioses (e.g., mycorrhizal networks) and reduce microbial diversity [89,90,91]. While organic amendment (e.g., compost and manure) may offer a more sustainable alternative [92,93], they tend to release nutrients more slowly, which can limit their immediate effectiveness in karst ecosystem restoration. A combined application of both organic and chemical fertilizers may offer a more balanced and effective approach, improving both plant growth and soil microbial community stability [92,93,94].

The leaching of chemical nutrients, rather than runoff, is particularly problematic in karst ecosystems due to their high permeability, which allows nutrients to quickly reach groundwater systems and contaminate local water sources [95]. This nutrient leaching poses significant ecological risks, including the eutrophication of waters, toxic algal blooms, low dissolved oxygen, and loss of aquatic biodiversity [96,97,98]. Water availability plays a critical role in nutrient cycling and microbial community dynamics in these ecosystems. However, excessive water can lead to nutrient leaching, which may exacerbate eutrophication. Moderate precipitation can dilute chemical nutrient concentrations, mitigating microbial stress without exacerbating eutrophication risks [99,100]. In addition, ecological restoration strategies should not rely solely on nutrient supplementation but must integrate water retention measures (e.g., vegetation cover improvement) to reduce rapid infiltration [9]. The successful mitigation of eutrophication requires comprehensive management strategies that address nutrient inputs and water retention in tandem to avoid excessive nutrient build-up and water contamination [101,102]. Although our study did not incorporate nutrient and precipitation gradients, future research should address these variables to provide a more nuanced understanding of their effects. This will be crucial for developing targeted policies for vegetation restoration in karst landscapes and guiding effective ecological management strategies.

## 5. Conclusions

This study highlights the critical role of water availability in regulating soil microbial communities and enhancing their stability under nutrient addition in karst shrublands. Nitrogen and phosphorous additions, whether applied individually or in combination, tended to reduce microbial network complexity and stability. In contrast, the water addition improved network complexity and stability and mitigated the negative effects associated with nutrient inputs, with the combined application of water and phosphorous showing the most pronounced positive effects. Water addition led to an increased soil microbial abundance, and nitrogen and phosphorous additions increased soil fungal diversity. The interaction between water and single nutrients supports a higher soil microbial co-occurrence network complexity and stability. Soil pH, available phosphorous, the ratio of carbon to phosphorous, and ammonium nitrogen were identified as key factors influencing microbial abundance, diversity, composition, and network complexity. These findings provide valuable guidance for improving restoration practices in ecologically fragile karst landscapes by emphasizing the importance of managing water availability alongside nutrient inputs. We acknowledge that this study is based on a single sampling campaign within one year and thus primarily reflects short-term microbial responses. Longer-term and multi-season monitoring would be valuable to confirm whether the patterns observed here persist over time and under varying climatic conditions. Future research should therefore extend to multi-year experiments to capture long-term dynamic changes in karst shrubland restoration under combined water and nutrient management. Additionally, incorporating metagenomic analysis and enzyme activity measurements in future studies would provide deeper insights into the functional potential of microbial communities and their role in ecosystem processes, such as nutrient cycling and microbial functional diversity.

## Figures and Tables

**Table 1:** Soil physico-chemical properties as affected by eight treatments. C, control; W, water addition; N, nitrogen addition; P, phosphorus addition; WN, water and nitrogen addition; WP, water and phosphorus addition; NP, nitrogen and phosphorus addition; and WNP, water, nitrogen, and phosphorus addition.

Variables	Treatments							
	C	W	N	P	WN	WP	NP	WNP
SWC	46.6 ± 2.5	53.3 ± 1.3	47.0 ± 1.0	50.9 ± 3.2	50.4 ± 3.1	51.5 ± 0.9	49.1 ± 1.4	53.2 ± 1.4
pH	7.5 ± 0.2	7.3 ± 0.2	7.4 ± 0.2	7.5 ± 0.2	7.1 ± 0.2	7.3 ± 0.2	7.1 ± 0.2	7.0 ± 0.1
SOC	53.4 ± 5.1	55.6 ± 4.9	48.1 ± 3.0	56.1 ± 6.0	50.0 ± 5.6	51.1 ± 5.5	50.4 ± 1.2	55.6 ± 3.9
TN	4.9 ± 0.3	5.1 ± 0.7	6.2 ± 1.8	5.6 ± 0.5	4.7 ± 0.5	4.5 ± 0.4	3.8 ± 0.4	4.4 ± 0.4
TP	1.9 ± 0.0	1.8 ± 0.2	1.6 ± 0.2	2.2 ± 0.2	1.9 ± 0.3	1.8 ± 0.2	1.8 ± 0.1	1.8 ± 0.1
TK	4.0 ± 0.6	4.4 ± 0.5	4.4 ± 0.4	4.0 ± 0.4	4.6 ± 0.4	4.6 ± 0.5	4.3 ± 0.4	5.0 ± 0.5
C:N	11.0 ± 1.1 bc	11.2 ± 0.7 ab	9.1 ± 1.2 c	10.0 ± 0.7 c	10.6 ± 0.6 bc	11.5 ± 0.5 abc	14.0 ± 1.6 a	13.1 ± 1.3 ab
C:P	28.8 ± 2.8	31.8 ± 1.3	30.1 ± 1.2	27.4 ± 4.2	27.5 ± 2.4	29.2 ± 1.9	28.9 ± 1.1	31.5 ± 1.1
N:P	2.6 ± 0.1	2.9 ± 0.1	3.6 ± 0.6	2.7 ± 0.3	2.6 ± 0.2	2.6 ± 0.3	2.2 ± 0.3	2.5 ± 0.2
NH_4_^+^	11.5 ± 1.2	11.5 ± 1.2	9.6 ± 0.6	11.5 ± 0.9	11.0 ± 0.7	11.1 ± 1.2	9.6 ± 0.6	12.1 ± 1.0
NO_3_^−^	16.3 ± 1.9 bc	16.6 ± 1.6 bc	25.8 ± 2.3 ab	20.3 ± 6.1 bc	32.6 ± 6.4 a	14.8 ± 1.1 c	23.8 ± 1.8 abc	25.0 ± 4.3 abc
AP	4.3 ± 0.6	3.0 ± 0.5	3.3 ± 0.8	4.3 ± 0.2	3.6 ± 0.7	3.3 ± 0.7	4.4 ± 0.2	4.3 ± 0.5
AK	108.4 ± 3.0	108.6 ± 4.5	98.9 ± 4.7	112.4 ± 6.0	103.1 ± 4.6	108.8 ± 6.8	97.2 ± 3.7	107.8 ± 6.1
Ca^2+^	3.1 ± 0.3	2.9 ± 0.3	2.7 ± 0.2	3.3 ± 0.3	2.7 ± 0.2	2.6 ± 0.3	2.7 ± 0.2	2.7 ± 0.2
Mg^2+^	3.3 ± 0.0	3.0 ± 0.2	3.0 ± 0.2	3.6 ± 0.2	2.8 ± 0.3	2.8 ± 0.3	3.0 ± 0.2	2.9 ± 0.3

SWC, soil water content, %; pH, soil pH; SOC, soil organic carbon, g kg^−1^; TN, soil total nitrogen, g kg^−1^; TP, soil total phosphorus, g kg^−1^; TK, soil total potassium, g kg^−1^; C:N, soil carbon to nitrogen ratio; C:P, soil carbon to phosphorus ratio; N:P, soil nitrogen to phosphorus ratio; NH_4_^+^, soil ammonium nitrogen, mg kg^−1^; NO_3_^−^, soil nitrate nitrogen, mg kg^−1^; AP, soil available phosphorus, mg kg^−1^; AK, soil available potassium, mg kg^−1^; Ca^2+^, exchangeable calcium, g kg^−1^; and Mg^2+^, exchangeable magnesium, g kg^−1^. Values are means ± SE (n = 5). Different letters within the same row denote significant differences among treatments at *p* < 0.05. No letter indicates no significant differences between treatments.

## Data Availability

The data used to support the findings of this study can be made available by the corresponding author upon request.

## Supplementary Materials

The following supporting information can be downloaded at: https://www.mdpi.com/article/10.3390/microorganisms13092012/s1, Figure S1: Discrimination of bacterial (a) and fungal (b) community compositions between different treatments based on linear discriminant analysis effect size (LEfSe). Bacterial and fungal lineages are relatively more abundant in different treatments, as indicated in different colors; Table S1: Effects of water, nitrogen, and phosphorus additions and their interactions on soil physico-chemical properties. Table S2: Effects of different treatments on soil physico-chemical properties.
