# Supplementary material for: Precipitation Alleviates Adverse Effects of Nitrogen and Phosphorus Enrichment on Soil Microbial Co-Occurrence Network Complexity and Stability in Karst Shrubland
Source: Microorganisms. 2025 Aug 28;13(9):2012. doi: 10.3390/microorganisms13092012 (PMC12471630; doi:10.3390/microorganisms13092012)

Supplementary Information

Jiangnan Li [1, 2, 3], Jie Zhao [2, 3, 4, *], Xionghui Liao [2, 3], Xianwen Long [2, 3], Wenyu Wang [2, 3], Peilei Hu [2, 3], Wei Zhang [2, 3, 4], Kelin Wang [2, 3, *]

[1] College of Environment and Ecology, Hunan Agricultural University, Changsha 410128, China

[2] Institute of Subtropical Agriculture, Chinese Academy of Sciences, Changsha 410125, China

[3] Huanjiang Agriculture Ecosystem Observation and Research Station of Guangxi, Guangxi Key Laboratory of Karst Ecological Processes and Services, Huanjiang Observation and Research Station for Karst Ecosystems, Chinese Academy of Sciences, Huanjiang 547100, Guangxi, China

[4] Guangxi Industrial Technology Research Institute for Karst Rocky Desertification Control, Nanning 530012, China

[*] Corresponding author and reprint request: Dr. Jie Zhao and Prof. Kelin Wang

Email for Jie Zhao: jzhao@isa.ac.cn

Email for Kelin Wang: kelin@isa.ac.cn

Tel.: +86-731-84619720

Fax: +86-731-84612685

This file includes:

Table S1 and Figure S1

Table S1 Effects of water, nitrogen, and phosphorus addition and their interactions on soil physico-chemical properties. C, control; W, water addition; N, nitrogen addition; P, phosphorus addition; WN, water and nitrogen addition; WP, water and phosphorus addition; NP, nitrogen and phosphorus addition; WNP, water, nitrogen, and phosphorus addition.

Variables	W		N		P		W×N		W×P		N×P		W×N×P	
	F	*p*	F	*p*	F	*p*	F	*p*	F	*p*	F	*p*	F	*p*
SWC	6.454	**0.016**	0.201	0.657	1.594	0.216	0.000	0.986	0.867	0.359	0.157	0.695	1.359	0.252
Soil pH	1.911	0.176	3.270	0.080	0.370	0.547	0.013	0.911	0.030	0.863	0.559	0.460	0.250	0.621
SOC	0.098	0.756	0.833	0.368	0.214	0.647	0.567	0.457	0.085	0.773	0.565	0.458	0.629	0.434
TN	0.718	0.403	0.187	0.668	1.667	0.206	0.001	0.970	0.139	0.712	1.769	0.193	2.503	0.123
TP	0.217	0.645	1.234	0.275	0.397	0.533	2.165	0.151	1.334	0.257	0.297	0.590	0.012	0.915
TK	2.151	0.152	0.998	0.325	0.229	0.635	0.008	0.927	0.274	0.604	0.026	0.873	0.047	0.830
C:N	0.658	0.423	1.151	0.291	5.341	**0.027**	0.150	0.701	0.205	0.654	7.685	**0.009**	1.632	0.211
C:P	0.559	0.460	0.015	0.902	0.025	0.876	0.549	0.464	0.396	0.534	1.154	0.291	1.020	0.320
N:P	0.548	0.464	0.024	0.879	4.213	**0.048**	0.945	0.338	1.444	0.238	2.351	0.135	3.828	0.059
NH_4^+	1.674	0.205	1.370	0.251	0.082	0.777	2.432	0.129	0.077	0.783	0.277	0.602	0.289	0.595
NO_3^-	0.075	0.786	13.714	**0.001**	0.477	0.495	1.532	0.225	1.174	0.287	1.261	0.270	0.000	0.983
AP	1.568	0.220	0.189	0.666	1.614	0.213	2.448	0.127	0.008	0.929	0.953	0.336	0.106	0.747

AK	0.636	0.431	4.741	**0.037**	0.253	0.618	1.601	0.215	0.030	0.863	0.008	0.930	0.506	0.482
Ca^{2+}	1.921	0.175	2.849	0.101	0.099	0.755	1.866	0.181	0.206	0.653	0.025	0.876	0.511	0.480
Mg^{2+}	3.955	0.055	2.232	0.145	0.155	0.696	1.171	0.287	0.524	0.475	0.032	0.859	0.548	0.464

SWC, soil water content, %; SOC, soil organic carbon, g kg^{-1}; TN, soil total nitrogen, g kg^{-1}; TP, soil total phosphorus, g kg^{-1}; TK, soil total potassium, g kg^{-1}; C:N, soil carbon to nitrogen ratio; C:P, soil carbon to phosphorus ratio; N:P, soil nitrogen to phosphorus ratio; NH_4^+, soil ammonium nitrogen, mg kg^{-1}; NO_3^-, soil nitrate nitrogen, mg kg^{-1}; AP, soil available phosphorus, mg kg^{-1}; AK, soil available potassium, mg kg^{-1}; Ca^{2+}, exchangeable calcium, g kg^{-1}; Mg^{2+}, exchangeable magnesium, g kg^{-1}.

Table S2 Effects of different treatments on soil physico-chemical properties.

Variables	Treatment	
	F	*p*
SWC	1.159	0.196
Soil pH	0.915	0.508
SOC	0.427	0.878
TN	0.998	0.451
TP	0.808	0.587
TK	0.533	0.803
**C:N**	**2.403**	**0.043**
C:P	0.531	0.804
N:P	1.908	0.101
NH_4^+	0.886	0.529
**NO_3^-**	**2.605**	**0.030**
AP	0.984	0.460
AK	1.111	0.380
Ca^{2+}	1.068	0.406
Mg^{2+}	1.231	0.315

SWC, soil water content, %; SOC, soil organic carbon, g kg^{-1}; TN, soil total nitrogen, g kg^{-1}; TP, soil total phosphorus, g kg^{-1}; TK, soil total potassium, g kg^{-1}; C:N, soil carbon to nitrogen ratio; C:P, soil carbon to phosphorus ratio; N:P, soil nitrogen to phosphorus ratio; NH_4^+, soil ammonium nitrogen, mg kg^{-1}; NO_3^-, soil nitrate nitrogen, mg kg^{-1}; AP, soil available phosphorus, mg kg^{-1}; AK, soil available potassium, mg kg^{-1}; Ca^{2+}, exchangeable calcium, g kg^{-1}; Mg^{2+}, exchangeable magnesium, g kg^{-1}.

Figure S1 Discrimination of bacterial (a) and fungal (b) community compositions between different treatments based on linear discriminant analysis effect size (LEfSe). Bacterial and fungal lineages are relatively more abundant in different treatments, as indicated in different colors.

(a)

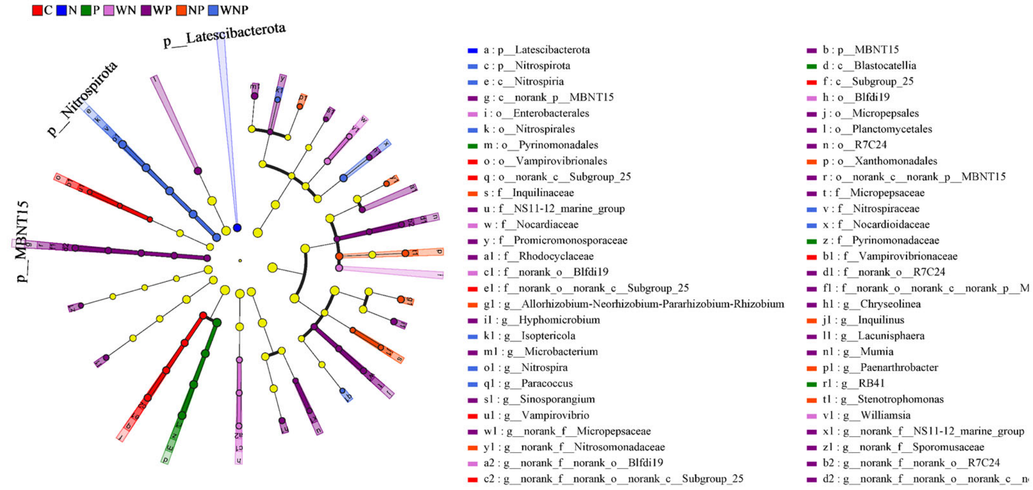

(b)

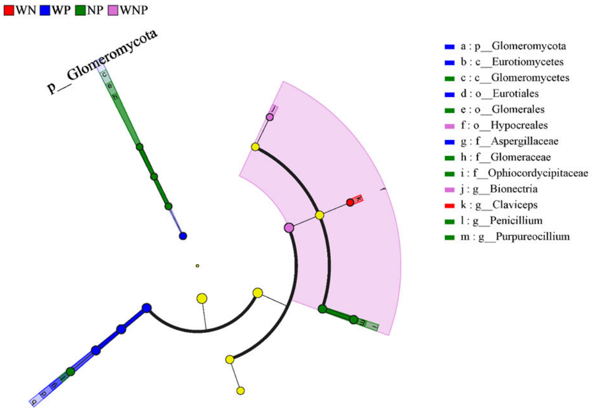

Supplement: Supplementary file 1 [file microorganisms-13-02012-s001.zip › microorganisms-3792449-supplementary.pdf]
